# Gray Platelet Syndrome in a Neonate With VACTERL Association: A Novel Homozygous Pathogenic Variant c.5257C>T in the NBEAL2 Gene

**DOI:** 10.7759/cureus.48359

**Published:** 2023-11-06

**Authors:** Badriah G Alasmari, Syed Rayees, Sami Althubaiti, Lina Elzubair, Safa Chendeb

**Affiliations:** 1 Department of Pediatrics, Armed Forces Hospital Southern Region, Khamis Mushait, SAU; 2 Department of Pediatric Oncology, King Abdullah International Medical Research Center, Jeddah, SAU; 3 Department of Pediatrics, King Abdulaziz Medical City, Ministry of National Guard Health Affairs, Jeddah, SAU; 4 Department of Pathology, Armed Forces Hospital Southern Region, Khamis Mushait, SAU

**Keywords:** nbeal2, novel, whole exome sequencing, homozygous, vacterl association, grey platelet syndrome

## Abstract

Gray platelet syndrome is a rare hereditary autosomal recessive condition distinguished by a mild to moderate propensity toward bleeding, moderate reduction in platelet count, and a significant decrease or complete absence of platelet alpha granules. VACTERL association is a condition of specific birth defects affecting multiple organ systems, with an unknown etiology. The acronym stands for vertebral anomalies (V), anal anomalies (A), cardiac anomalies (C), tracheoesophageal fistula (TE), renal anomalies or radial bone anomalies (R), and limb defects (L). To diagnose the VACTERL association, at least three of the aforementioned abnormalities should be present. This case report concerns a neonate born with a left absent thumb, a hypoplastic right thumb, an imperforate anus, and an atrial septal defect. During postoperative investigations, after addressing an anorectal malformation, the patient was found to have moderate thrombocytopenia and large gray platelets upon examination of a peripheral blood smear. A genetic analysis validated the pathogenic homozygous mutation c.5257C>T in the NBEAL2 gene, which corresponds to gray platelet syndrome.

## Introduction

Gray platelet syndrome (GPS), also referred to as "alpha reservoir disease," is a rare autosomal recessive hereditary disorder that affects the alpha granules of platelets and the proteins they contain [1]. The prevalence of this disease is extremely low, with less than one in 1,000,000 individuals being affected. Although the age at which diagnosis occurs can vary, symptoms typically manifest during the neonatal or early childhood period [2]. Electron microscopy is considered the most accurate diagnostic test for this condition. Due to the limited number of reported cases, there is currently no standardized management algorithm for GPS. Therefore, treatment decisions are based on the discretion of the physician and the patient's clinical condition [3]. VACTERL (vertebral anomalies, anal anomalies, cardiac anomalies, tracheoesophageal fistula, renal anomalies or radial bone anomalies, and limb defects) association is a non-random co-occurrence of multiple congenital malformations that occur sporadically without a clear underlying cause. The estimated incidence of this condition ranges from one in 10,000 to one in 40,000 live-born infants. Diagnosis is typically made based on the clinical presentation observed at birth [4].

## Case presentation

This report describes the case of a male neonate born to consanguineous parents via spontaneous vaginal delivery at term. The neonate had an Apgar score of 8 and 9 at one and five minutes, respectively, and weighed 2.6 kg at birth. The mother had a normal and uneventful pregnancy with no medication use and normal antenatal scans and ultrasound Doppler. There was no family history of congenital abnormalities or hematological disorders. The parents had one healthy child prior to this baby.

**Figure 1:**
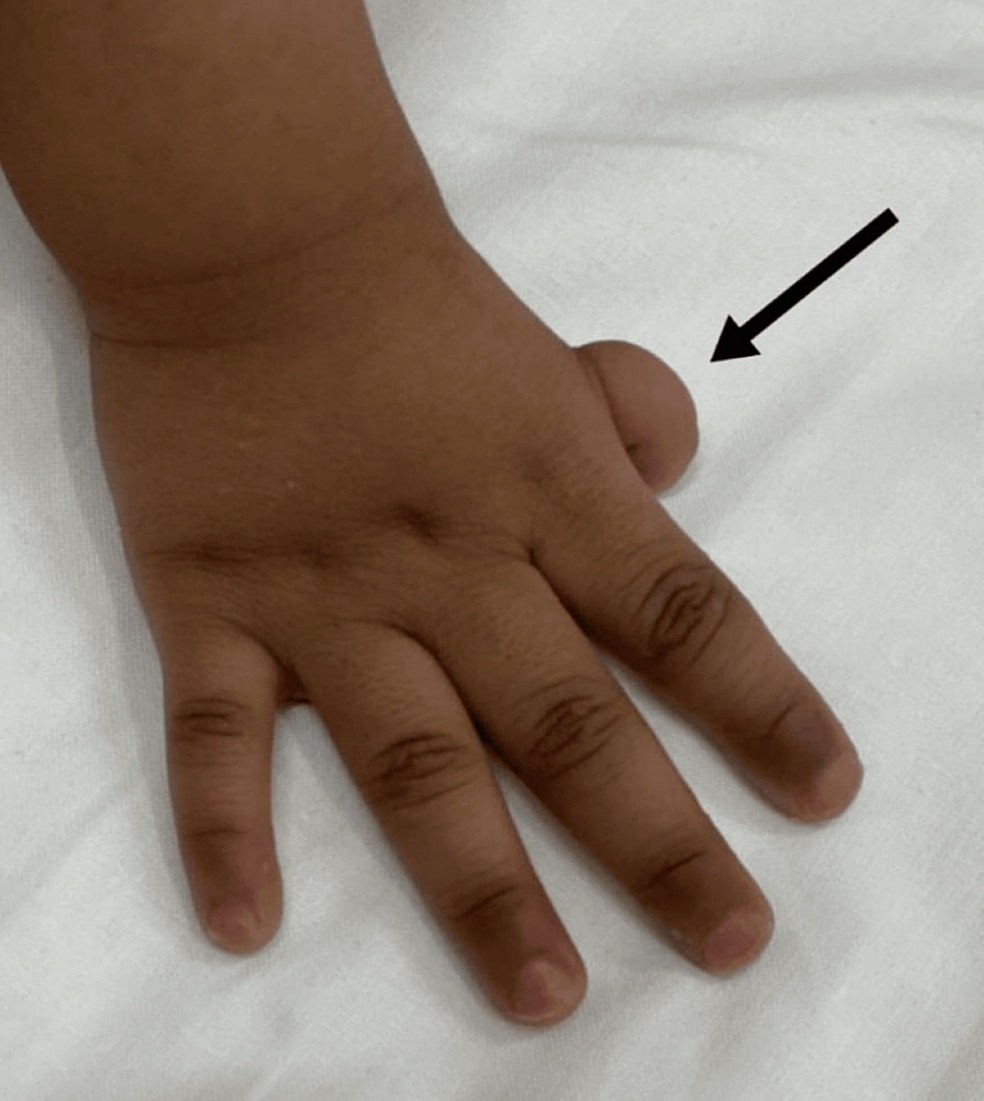
Arrowhead showing hypoplasia of the right thumb.

**Figure 2:**
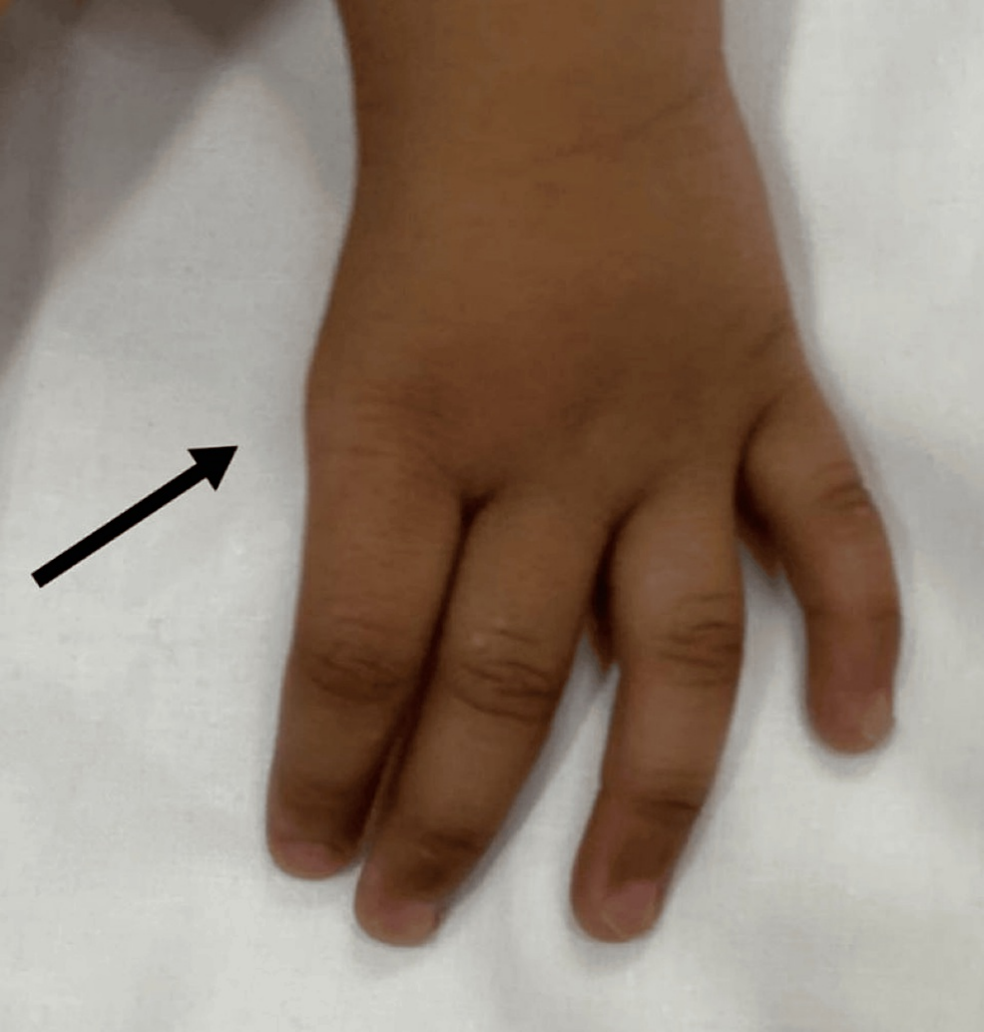
Arrowhead showing aplasia of the left thumb.

Upon physical examination, the neonate exhibited dysmorphic features such as a depressed nasal bridge, a high-arched palate, a hypoplastic right thumb (Figure 1), an absent left thumb (Figure 2), and an imperforate anus. The chest examination revealed equal air entry bilaterally, while the cardiovascular examination showed S1+S2 with a systolic murmur in the left upper sternal border. The abdomen was found to be soft and non-tender, with no organomegaly present.

At birth, he received intramuscular vitamin K. However, at 12 hours old, he developed respiratory distress and was subsequently transferred to the neonatal intensive care unit (NICU). While in the NICU, the neonate received oxygen through a nasal cannula and was gradually weaned off of it. He had an imperforate anus with a recto-urethral fistula, which required a diversion colostomy to be performed on the third day of life. The postoperative investigations revealed moderate thrombocytopenia with normal hemoglobin and white cell counts (Table 1). On day five of life, he developed petechiae over the back, chest, and near the cannula site with no bleeding manifestation.

**Table 1:** Complete blood count of the patient.

Measured entity	Postoperative value	Normal range
Hemoglobin	13.4 g/dl	10.9-15 g/dl
Platelets	80 units 10^9/L	150-400 units 10^9/L
White blood cell	8.58 units 10^9/L	9-30 units 10^9/L

**Figure 3:**
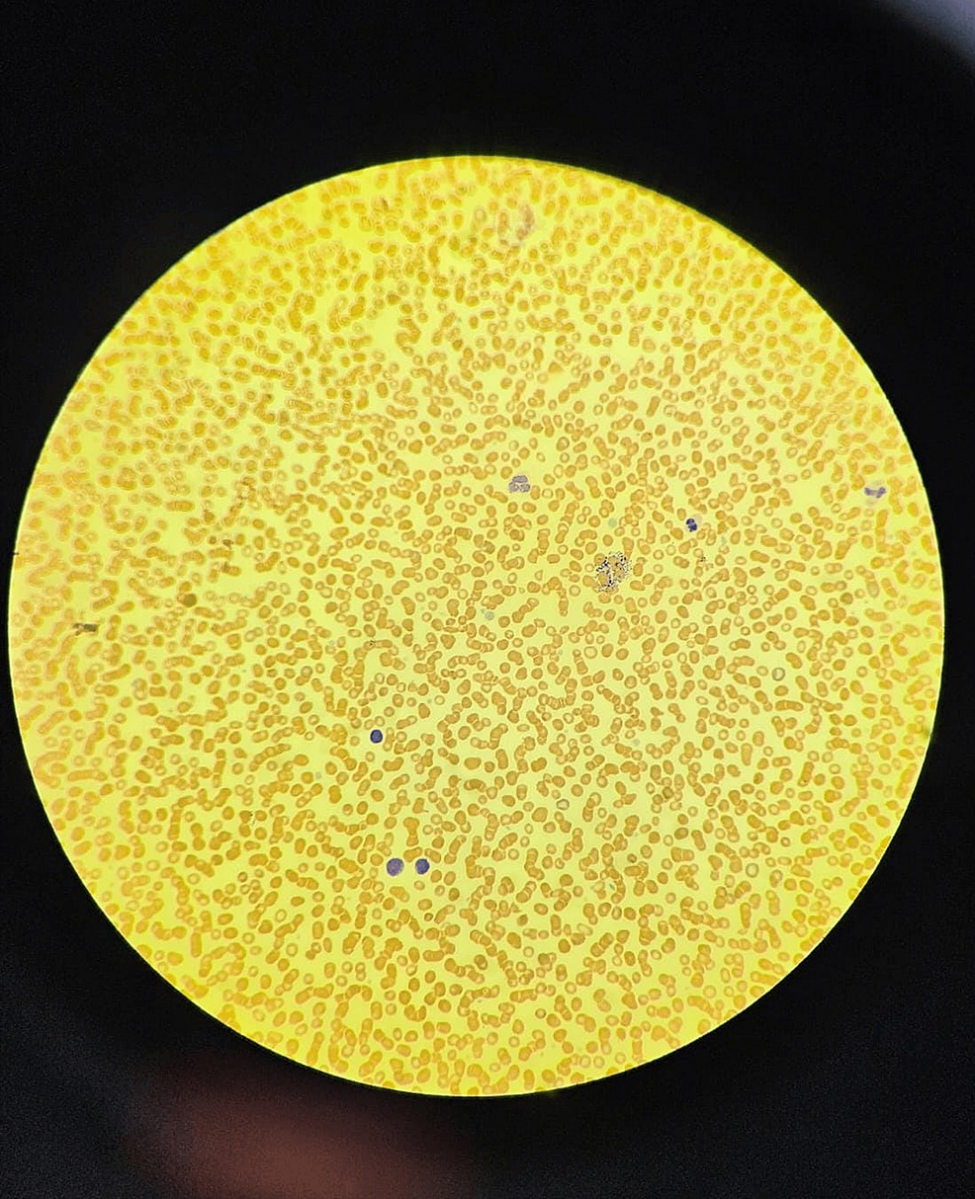
Peripheral blood smear (20x with Wright-Giemsa stain) showing hypochromic microcytic red blood cells, occasional polychromasia, moderate thrombocytopenia with large platelets, and manual platelets count of 60 x 10^9/L.

**Figure 4:**
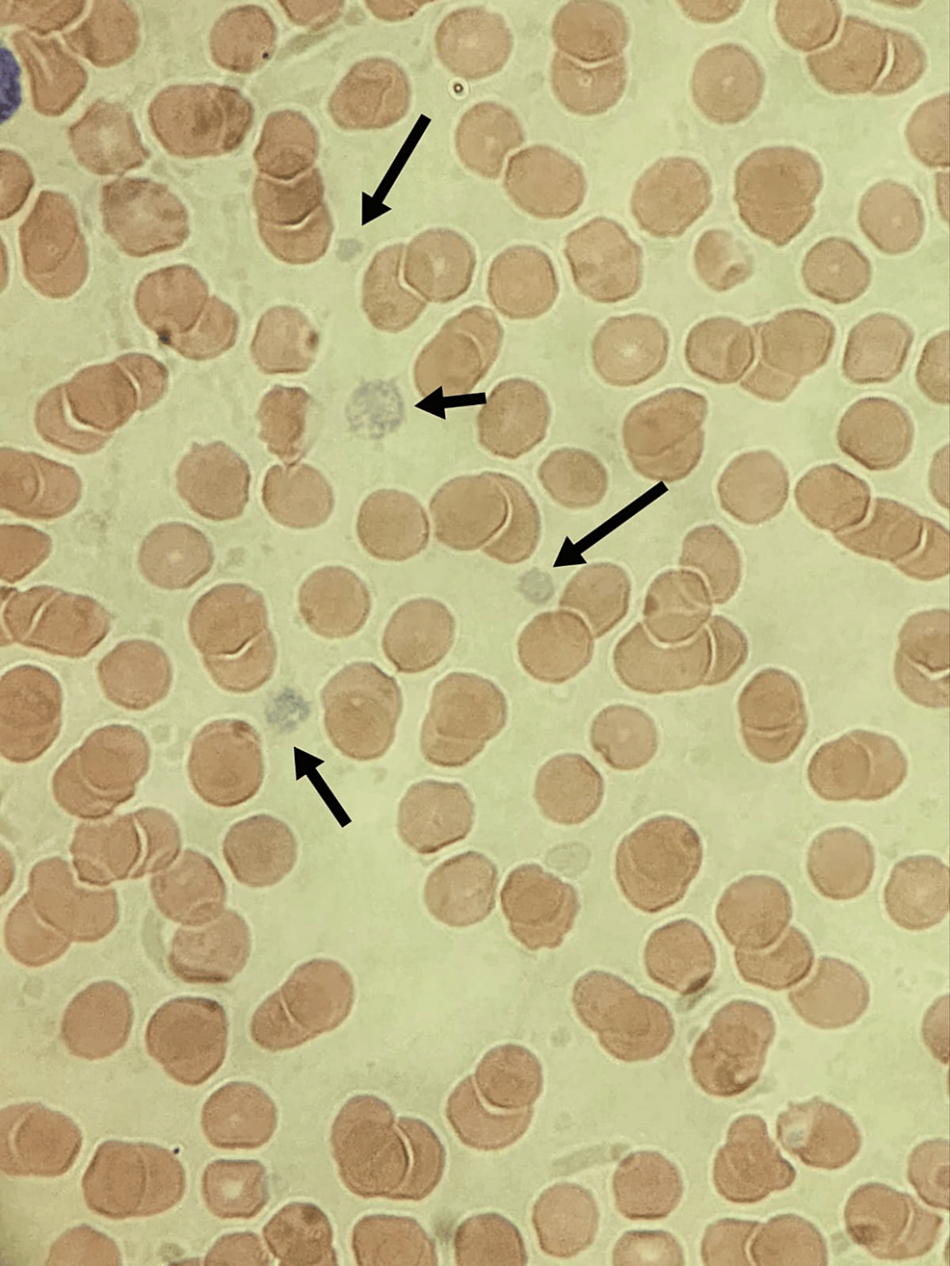
Peripheral blood smear (100x magnification with Wright-Giemsa stain) showing moderate thrombocytopenia with prominent large gray platelets.

Peripheral blood smear showed occasional large gray platelets with thrombocytopenia (Figures 3, 4). To ascertain the underlying cause of thrombocytopenia, further investigations were conducted. Coagulation profile, liver function test, renal profile, thyroid function test, and newborn screening all yielded results within the normal ranges. Blood and urine cultures displayed no signs of growth. TORCH (toxoplasmosis, other agents, rubella, cytomegalovirus, and herpes simplex) screen was negative. Ultrasounds of the brain and abdomen revealed no abnormality. An echocardiogram revealed secundum atrial septal defect. Chromosomal breakage analysis was negative for Fanconi anemia. To explore any potential association between thrombocytopenia and VACTERL, a genetic study was conducted. The whole exome sequencing (WES) study identified the homozygous variant c.5257C>T in the NBEAL2 gene as causative for the autosomal recessive GPS (Table 2); however, it could not explain the simultaneous presence of VACTERL association with GPS.

**Table 2:** The performed analysis identified the homozygous variant in the NBEAL2 gene (OMIM: *614169). This variant leads to a premature stop codon, and subsequent mRNA degradation (nonsense-mediated decay) or truncation of the protein. This variant is classified as likely pathogenic. MIM: Mendelian Inheritance in Man; OMIM: Online Mendelian Inheritance in Man.

Gene (isoform)	Phenotype MIM number	Variant	Zygosity	MAF gnomAD (%)	Classification
NBEAL2 (NM_015175.3)	139090 (AR)	c.5257C>T, p.(Gln1753*) chr3:47043966	Homozygous	0	Likely pathogenic

During his hospitalization, the patient was under the regular care of a multidisciplinary team consisting of a neonatologist, a hematologist, a pediatric surgeon, and a geneticist.

On the 20th day of life, the patient was discharged in a stable condition with a colostomy in place and scheduled for regular follow-up appointments. Throughout the follow-up period, the patient's platelet count remained stable, with no signs of bleeding, and the child exhibited satisfactory growth.

At the age of one year, the patient was admitted for an elective procedure to close the colostomy and form an anoplasty. Following the successful procedure, the patient was discharged home. At the time of discharge, the patient’s physical development was appropriate for his age. His recent laboratory investigations as of June 2023 revealed hemoglobin of 13 g/dl, white blood cells of 11.2 units 10^9/L, and platelets of 95 units 10^9/L.

## Discussion

GPS is an exceptionally rare inherited autosomal recessive disease affecting the alpha-granules in platelets. It is characterized by macro-thrombocytopenia, which causes mild to moderate bleeding, splenomegaly, and myelofibrosis. In GPS, there is a reduction or complete absence of alpha granules within platelets, leading to the release of proteins that are typically contained within them into the bone marrow [1]. The NBEAL2 (neurobeachin-like 2) gene, located on chromosome 3p, is the gene that undergoes mutation in GPS. The primary function of alpha granules is to store proteins that promote platelet adhesiveness and facilitate wound healing when activated during an injury [2]. Symptoms of GPS typically manifest in infancy or childhood and include prolonged bleeding, epistaxis, and easy bruising. In adolescent girls, it may present as menorrhagia. The absence of alpha granules causes the release of hemostatic proteins, including growth factors, into the bone marrow, resulting in myelofibrosis. This, in turn, leads to splenomegaly later in life due to extramedullary hematopoiesis [3]. GPS patients often exhibit elevated serum B12 levels, although the exact cause of this elevation remains unknown [5]. Electron microscopy is the recommended diagnostic test for GPS. A Wright-stained peripheral smear reveals the presence of pale or gray (hypogranular) colored platelets. Additionally, diagnosis can be confirmed through WES studies [6]. The size of the platelets can guide further investigation in cases of thrombocytopenia. By considering the patient's clinical history, we can determine whether thrombocytopenia is acquired or inherited. Acquired forms are more prevalent in children. GPS is associated with a decreased aggregation response to collagen and thrombin [2].

VACTERL association is a cluster of congenital malformations with at least three of the following anomalies present: vertebral anomalies with or without rib anomalies (60-80% of patients), anal anomalies (50-90%), cardiac defects (40-80%), tracheoesophageal fistula with or without esophageal atresia (50-80%), renal or radial anomalies (50-80%), and limb defects (40-50%). This condition is typically identified at birth or in the initial days of life [4]. The etiology of the VACTERL association is currently unknown, and almost all cases are sporadic with no identified inherited cause. It has been reported to occur in <1-9/100,000 infants, and the annual incidence is 1/10,000 to 1/40,000 live births [7].

The vertebral anomalies include hemivertebrae, butterfly vertebrae, wedge vertebrae, vertebral fusions, and supernumerary or absent vertebrae. The severity of these anomalies can vary widely among affected patients. Anal anomalies include imperforate anus or anal atresia. Anal stenosis may present later with signs of obstruction. Genito-urinary anomalies are common in patients with anorectal malformations. Cardiac anomalies include structural heart defects. Renal anomalies include renal agenesis, horseshoe kidney, and cystic and/or dysplastic kidneys. Limb anomalies include thumb hypoplasia/aplasia, radial anomalies, polydactyly, and lower limb anomalies. Recently, the presence of a single umbilical artery has been observed in VACTERL patients, which can aid in the antenatal diagnosis of this condition [4]. Management of the VACTERL association typically involves surgical correction of the respective anomaly and follow-up for any complications post surgery. Despite the presence of multiple congenital malformations, VACTERL babies are developmentally and intellectually normal [7].

Currently, there is no specific management or treatment algorithm for GPS due to the limited number of affected cases. Generally, the approach involves anticipating and preventing bleeding episodes. A platelet count below 30,000/microliter may necessitate splenectomy. Desmopressin (DDAVP) may be utilized as a hemostatic agent [8].

In this particular case, the patient presented with an imperforate anus and a recto-vesical fistula, as well as upper limb anomalies. During routine investigation after surgical correction of the anorectal malformation, thrombocytopenia was unexpectedly discovered. Subsequent investigation was conducted to explore the potential relationship between thrombocytopenia and VACTERL, utilizing the WES study. The results of the WES study revealed that the patient had GPS, specifically in a homozygous state. Notably, this homozygous variant has not been previously reported in the literature or the internal database (HGMD 2021.3). The frequency of this variant in the general population remains undocumented (gnomAD v2.1.1 controls). Therefore, it can be concluded that the homozygous pathogenic variant c.5257C>T in the NBEAL2 gene is responsible for the autosomal recessive GPS. However, it is important to note that this variant does not account for the co-occurrence of GPS and VACTERL in the same patient.

Through a proactive multidisciplinary approach to management, the patient has experienced no significant complications thus far and is progressing well in terms of development. The management of GPS is not specific. Given that it is an autosomal recessive disease, targeted molecular genetic testing can be offered to family members of the patient.

## Conclusions

GPS is an exceedingly rare inherited disorder. Our patient was born with VACTERL association and was subsequently diagnosed with GPS, although no clear correlation between the two conditions could be established. It is imperative that these patients receive regular follow-up care in a clinical setting to monitor for any potential long-term complications, and a tailored management strategy should be devised specifically for their unique needs. To facilitate the antenatal diagnosis of VACTERL, meticulous antenatal scans should be conducted at primary healthcare centers, enabling the prompt referral of such cases to specialized maternity and neonatal healthcare facilities.
